# Wild birds as potential reservoirs of antimicrobial-resistant *Escherichia coli*: a systematic review

**DOI:** 10.3389/fmicb.2025.1615826

**Published:** 2025-09-08

**Authors:** Catherine W. Mbuthia, Abubakar S. Hoza

**Affiliations:** ^1^Department of Veterinary Microbiology, Parasitology and Biotechnology, College of Veterinary Medicine and Biomedical Sciences, Sokoine University of Agriculture, Morogoro, Tanzania; ^2^Southern African Centre for Infectious Disease Surveillance (SACIDS) Foundation for One Health, Sokoine University of Agriculture, Morogoro, Tanzania

**Keywords:** AMR, CIAs, *E. coli*, ESBL, MDR, One Health, Wild birds

## Abstract

Antimicrobial resistance (AMR) is currently a major global problem affecting humans, animals, and the environment. The role of wild birds in this epidemiological circuit has been the subject of several studies, but it is still far from being assessed. This review summarizes findings from 51 studies published between 2014 and 2024, examining resistant *Escherichia coli* (*E. coli*) from wild birds, with a focus on extended-spectrum beta-lactams (ESBLs) and other World Health Organization critically important antimicrobials for human medicine (WHO CIA List). The analysis reveals higher levels of AMR *E. coli* in wild birds in low and middle-income countries than in high-income countries (HICs). Particularly concerning is the high resistance observed to WHO CIA List: 100% resistance to cefotaxime, ceftazidime, nalidixic acid, gentamicin, and over 90% resistance to ciprofloxacin. Among the ESBL-producing *E. coli*, the genes coding for ESBLs (*^bla^*ESBL) were predominant (76.5%, 377/493). Key gaps in the existing studies include: limited understanding of the sources of AMR for wild birds, limited comparative analyses of AMR in wild birds and other One Health sectors, and minimal longitudinal and satellite-tracking or telemetry approaches to monitor the persistence and transboundary movements of AMR in these birds. To address this, we advocate using standardized sampling methods, longitudinal studies incorporating satellite tracking, and whole-genome sequencing to better elucidate the role of wild birds in the global dissemination of AMR. Additionally, we emphasize the need to strengthen AMR surveillance in wild birds improve data reporting, and implement robust environmental management strategies within the One Health context to mitigate AMR transmission by wild birds.

## Introduction

Antimicrobial resistance (AMR), often referred to as the “silent pandemic,” poses a severe threat to global public health (Laxminarayan, 2022). While AMR is a naturally occurring phenomenon, the widespread overuse and misuse of antimicrobials over the last 80 years have dramatically accelerated its emergence and spread (Christaki et al., 2020). Although AMR is a worldwide challenge affecting developed and developing countries, its prevalence differs significantly across regions (Frost et al., 2019). Reports show a rapid global increase in AMR and its relentless spread between countries (Aljeldah, 2022). The World Health Organization (WHO) has warned of a “post-antibiotic era” where common, previously treatable bacterial infections could become fatal (Shankar and Balasubramanium, 2014). This means that the impact of AMR on humans and animals is profound and complicated, leading to longer hospital stays, chronic infections, increased economic burdens, and increased mortality (Ferraz, 2024). If unchecked, AMR is projected to cause 10 million deaths by 2050 (O’Neill, 2016).

*Escherichia coli (E. coli)* is a key species for monitoring AMR globally (Anjum et al., 2021). As a member of the Enterobacteriaceae, *E. coli* has developed significant resistance to crucial broad-spectrum cephalosporins. This resistance is mainly due to plasmid-mediated extended-spectrum beta-lactamases (ESBLs) and both chromosomal and plasmid-encoded AmpC beta-lactamases (pAmpC), exacerbating the fight against AMR (Duggett et al., 2020). Interestingly, even though wild birds have limited direct exposure to antimicrobials, they have been identified as potential reservoirs of resistant *E. coli*. Often, these birds pick up resistant strains when foraging in polluted environments, like antimicrobial-treated fields (Blanco and Bautista, 2020), or contaminated surface water (Yuan et al., 2021). For instance, studies in Egypt found 100% genetic similarity between *mcr-1*-producing *E. coli* from a migratory bird and surface water collected from the same trapping sites (Ahmed et al., 2019). This suggests that wild birds, through their mobility and ecological adaptability, can acquire and spread AMR bacteria and genes to humans, livestock, and the environment.

To combat AMR, the WHO adopted a global action plan (GAP) in May 2015. The GAP comprised five interdependent objectives: (1) to improve awareness and understanding of AMR through effective communication, education and training; (2) to strengthen the knowledge and evidence base through surveillance and research; (3) to reduce the incidence of infection through effective sanitation, hygiene and infection prevention measures;(4) to optimize the use of antimicrobial medicines in human and animal health; and (5) to develop the economic case for sustainable investment that takes into account the needs of all countries and to increase investment in new medicines, diagnostic tools, vaccines and other interventions. The GAP objectives were adopted as national action plans (NAPs) by most countries in their fight against AMR (World Health Organization, 2015).

In 2020, the Quadripartite [the WHO, the Food and Agriculture Organization of the United Nations (FAO), the World Organization for Animal Health (OIE), and the United Nations Environment Programme (UNEP)] was formed. The Quadripartite urged the stakeholders, including policy-makers, technical staff, academics, researchers, members of civil society, private-sector representatives, development partners and donors, within member states to move from an early to a sustainable implementation phase through a sector-specific programme and a joint collaborative One Health approach at local, national, regional and global levels, guided by the 2015 GAP. To sustainably balance and optimize the health of humans, animals, plants, food production and environmental protection and ecosystem interfaces, the Quadripartite recommended (1) a robust governance structure to ensure accountability and acceleration of NAPs implementation in countries, (2) innovations to secure the future, (3) global collaborations for more effective action, (4) investments for a sustainable response and (5) to strengthen accountability and global governance. By 2021, about 84% of member states provided annual reports on the implementation of their multi-sectoral NAPs against AMR (World Health Organization, UNEP United Nations Environment Programme and World Organisation for Animal Health, 2023).

Despite their significant role in spreading AMR, wildlife, particularly wild birds, and the environment are often overlooked in health security strategies, which tend to primarily focus on humans, domestic animals, and plants. However, monitoring the carriage of resistant bacteria in wild birds should be a priority within the One Health approach to combat AMR (Guardia et al., 2024). Wild birds utilize eight major migratory routes globally, including the prominent East Atlantic, Black Sea-Mediterranean, and East Asia-East Africa flyways (Seleem et al., 2021). As birds traverse these routes, they can acquire and disseminate AMR bacteria and genes across international borders. Notably, Southeast Asia is a major hot spot for AMR in animals (Chua et al., 2021). Africa, serving as a major flyway for over two billion Palearctic migratory birds, plays a crucial role in the seasonal movement of these birds across the African-Eurasian flyway. This global movement makes migratory birds potential key contributors to the worldwide spread of AMR (Guilherme et al., 2023). The first AMR strains of *E. coli* in wildlife were isolated in pigeons in 1975 (Sato et al., 1978). Since then, numerous studies in various countries have recognized wild birds as potential reservoirs and disseminators of antimicrobial-resistant *E. coli.*

This systematic review examines the current data on the role of migratory and resident wild birds as potential reservoirs of resistant *E. coli* and its associated genes globally. It specifically focuses on resistance to ESBL and other WHO CIA List. The review further evaluates evidence suggesting wild birds as potential disseminators of resistant *E. coli*, especially in studies that genetically link resistant strains found in wild birds to those in other One Health sectors. The review analyzes resistance patterns by geographic locations, wild bird species, income-indexes of countries, socio-economic, behavioral, and political influences on the occurrence of AMR in wild birds, and evidence of phylogenetic relationships that indicate cross-border transmission or involvement in the broader quintessential One Health AMR matrix. Finally, based on the analyzed data, the review provides recommendations for integrating wild birds into the One Health strategies to combat AMR.

## Materials and methods

We performed a systematic review following the Preferred Reporting Items for Systematic Reviews (PRISMA; Page et al., 2021; Supplementary Table 1). The two authors defined the research questions, objectives, search strategy, and inclusion/exclusion criteria.

### Search strategy

A literature search was conducted to summarize available data on wild birds as potential reservoirs of antimicrobial-resistant *E. coli* across countries. The search focused on publications from 2014 to 2024 and included publisher databases such as PubMed, ProQuest, and Scopus, as well as gray literature from the Google Scholar search engine. The search terms included wild birds* as reservoirs* of antimicrobial resistance* OR antibiotic resistance* E. coli*. These searches were then refined by merging them with each of the following continents: Africa*, Europe*, Asia*, North America*, South America*, and Oceania*. Details of the search strategy are available in Supplementary Table 2.

### Eligibility criteria

We aimed to identify peer-reviewed data published from 2014 to 2024, with no geographical or sampling site limits, that investigated AMR *E. coli* in wild birds. We looked for studies on resistant *E. coli* and the associated genes in different wild bird species (categorized by their mobility and habitat, i.e., resident vs. migratory, and water birds vs. non-water birds). Thus, we included only studies providing at least one of the following information: (i) wild bird species where resistant (multi-drug, ESBL-producing or resistant to one or two antimicrobial classes) *E. coli* was recovered, (ii) wild birds that showed phenotypic resistance to the WHO CIA List (iii) wild birds that showed genotypic resistant *E. coli* to the WHO CIA List. Details of inclusion and exclusion criteria are provided in Supplementary Table 3.

### Identification and screening of articles

**FIGURE 1:**
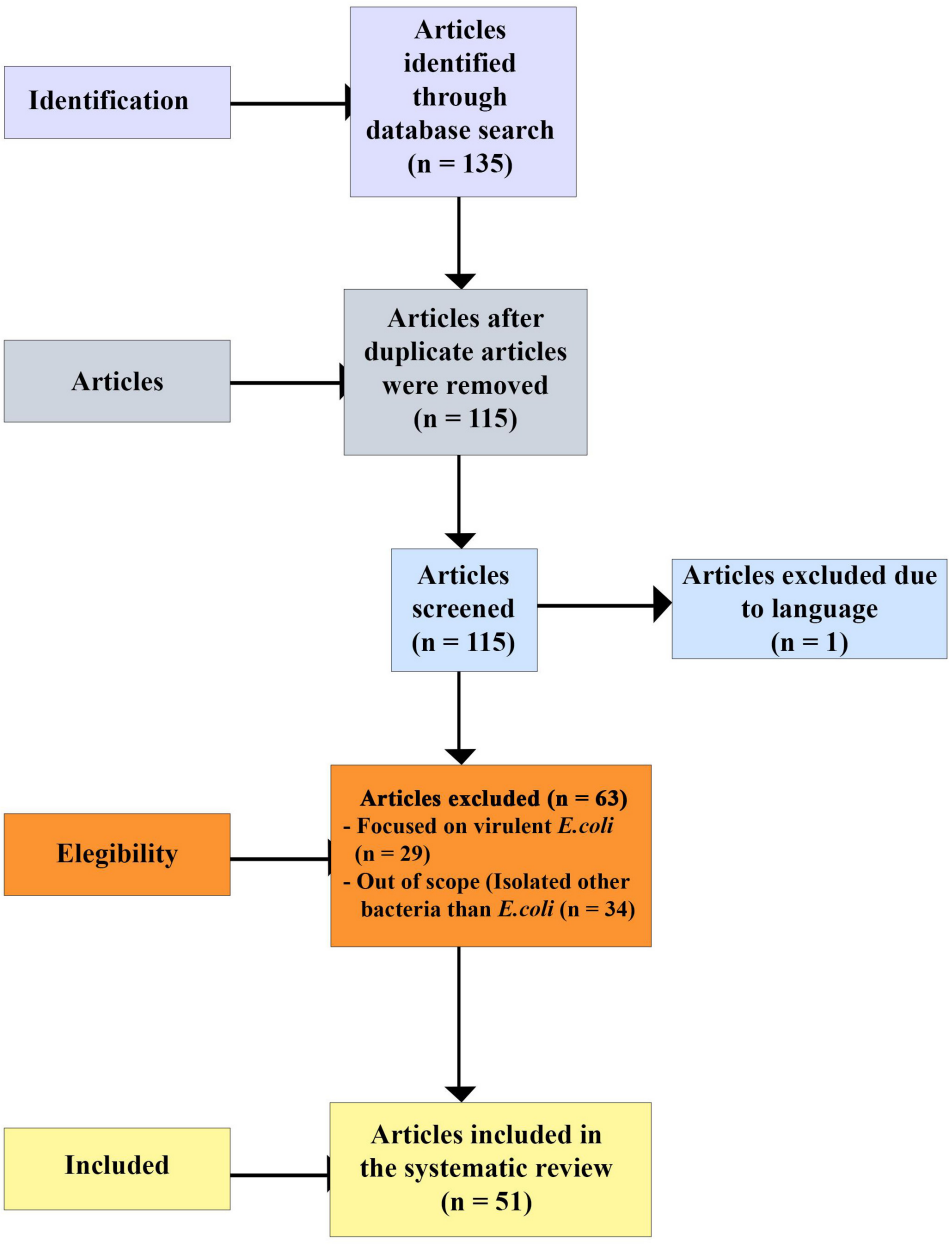
Article search flow diagram.

A total of 135 articles were identified. After removing 20 duplicates, 115 articles remained. A second screening was conducted based on language, where titles and abstracts were reviewed, leading to the exclusion of one article written in German. The remaining 114 articles were then evaluated for eligibility by reviewing their titles and abstracts for scopes and relevance. At this stage, 63 articles were excluded as they either did not focus on *E. coli* or only presented data on virulent *E. coli*. Ultimately, 51 articles met the eligibility criteria and were included in this review, as shown in Figure 1.

### Data extraction

Data were extracted from pertinent peer-reviewed publications that reported the proportions of resistant *E. coli* strains found in wild birds across different countries. The extracted information was summarized in a Microsoft Excel template (Supplementary Table 4). The file included the study continent, region and country, the World bank income category of each country, the avian mobility patterns (migratory or resident), the sample size, the avian species (water or non-water bird), the sample type collected, other sampled One Health sectors within the wild birds trapping sites, description of the sampling sites, suggested AMR origin in wild birds, the proportions of *E. coli*, antimicrobial susceptibility tests performed, the antimicrobials tested, the proportions of resistant *E. coli* [multi drug-resistant (MDR), ESBL-producing and resistant to one or two classes of antimicrobials], the molecular typing method used to characterize the resistant *E. coli* strains, the studies that characterized ESBL-producing *E. coli* strains and phylogenetic relationships. Phenotypic and molecular data of *E. coli* resistant to ESBLs and WHO CIA List, our major AMR patterns of interest, were represented in Supplementary Tables 5, 6.

## Results

### Geographic locations and wild birds studied for AMR *E. coli*

**FIGURE 2:**
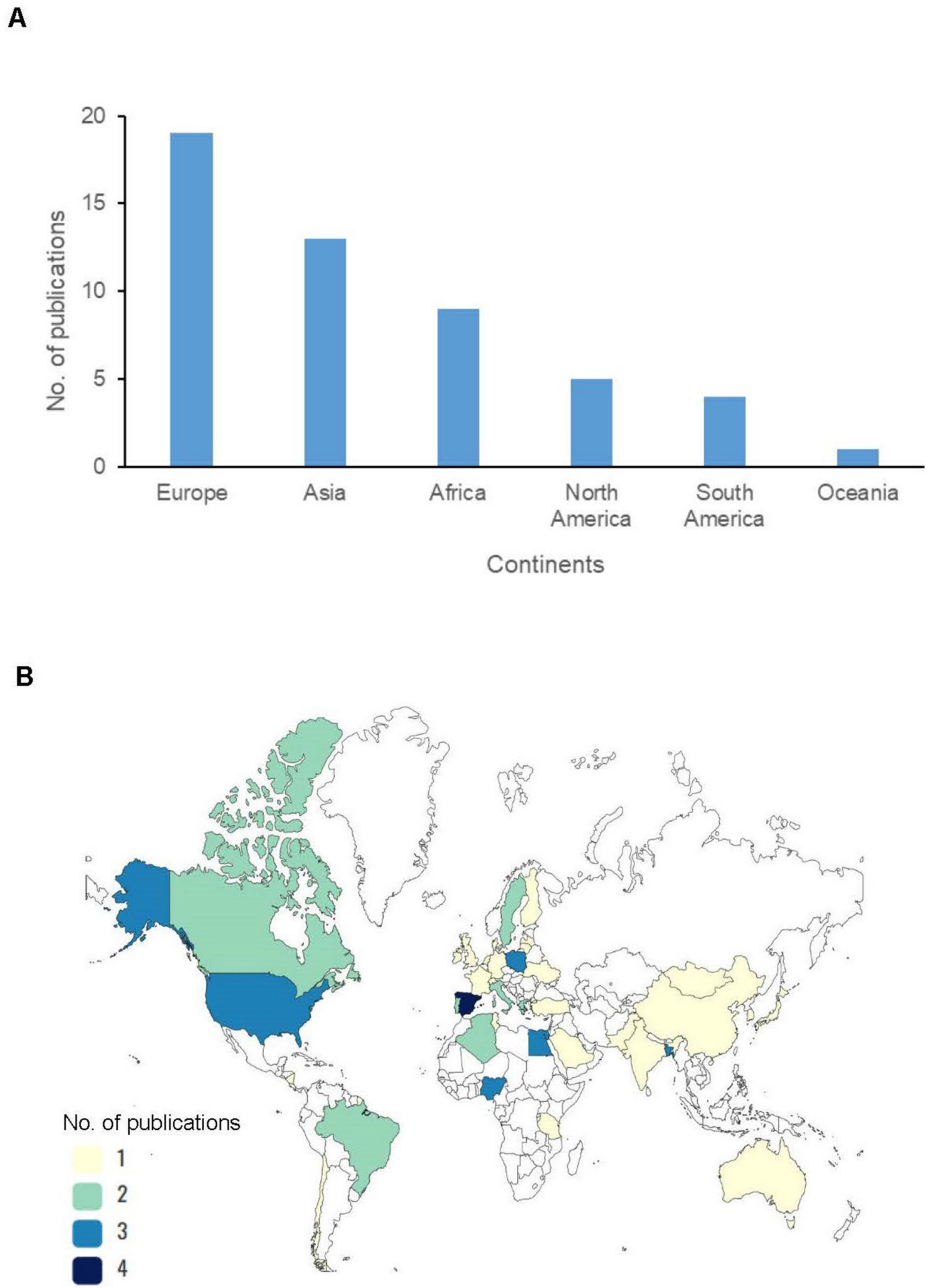
**(A)** Number of publications of antimicrobial resistance (AMR) *Escherichia coli* in wild birds per continent. **(B)** Number of publications of AMR *E. coli* in wild birds per country.

Studies on resistant *E. coli* from wild birds were carried out across the six continents. The majority of studies originated from Europe (37.2%, 19/51), followed by Asia (25.5%, 13/51) (Figure 2A). These studies spanned 39 countries, with high-income countries (HICs) such as Spain (Alcalá et al., 2016; Martín-Maldonado et al., 2022), the United States, and Poland (Nowaczek et al., 2021; Skarżyńska et al., 2021) having the highest number of publications (Figure 2B). In contrast, there were few publications from most low-and middle-income countries (LMICs), as Tanzania (Madoshi et al., 2021), Tunisia (Ben Yahia et al., 2018), Pakistan (Mohsin et al., 2017), and India (Raghav et al., 2022) (Supplementary Table 4). Notably, the number of publications from both HICs and LMICs did not correspond with the observed frequency of AMR *E. coli* isolates reported. Wild birds in LMICs (Chika et al., 2018; Islam et al., 2021, 2022) recorded more AMR *E. coli* than those in the HICs (Zurfluh et al., 2019; Athanasakopoulou et al., 2021, 2022; Eckenko et al., 2024) (Supplementary Table 4). An exception to this trend was observed in the United States of America (USA), where wild birds feeding from commercial feedlots exhibited exceptionally high levels of AMR *E. coli* (Chandler et al., 2020). The studies reviewed also differed in the avian species studied. According to mobility patterns, migratory species accounted for 57.4% (355/618) while resident species were 42.6% (263/618). Additionally, the studies prioritized non-water birds (terrestrial, arboreal, aerial) (57.2%, 308/538) over water bird species (42.8%, 230/538) (Supplementary Table 4).

### Phenotypic *E. coli* resistance patterns in wild birds to the WHO CIAs for human medicine

Phenotypic screening of resistant *E. coli* was conducted in 98% (50/51) of the studies using disk diffusion (72.5%, 37/51), Epsilometer tests (*E*-tests) (3.92%, 2/51), microbroth dilution (11.8%, 6/51), Vitek-2 system (9.8%, 5/51) and BD Phoenix system (2%, 1/51) (Supplementary Table 4). Given the high heterogeneity in susceptibility methods, antimicrobials tested, avian species, and sample size, a comparison of phenotypic AMR prevalence across studies could not be performed. However, the percentages of MDR-*E. coli*, ESBL-producing *E. coli*, and resistance to one or two antimicrobial classes were recorded for each study (Supplementary Table 4). Most studies reported diverse phenotypic resistance patterns to various WHO CIA List. Interestingly, most *E. coli* strains isolated from the wild birds showed susceptibility to antimicrobials used to treat multi-drug-resistant bacteria such as carbapenems, amikacins, and colistins (Supplementary Table 5).

### Molecular *E. coli* resistance patterns in wild birds to the WHO CIAs for human medicine

Antimicrobial resistant genes (ARGs) in *E. coli* isolates from wild birds were identified in 70.6% (36/51) of the studies using various molecular techniques (Supplementary Table 4). About 15.7% (8/51) of the studies used polymerase chain reaction (PCR) alone, or in combination with sequencing (39.2%, 20/51). Whole genome sequencing (WGS) was applied in 23.5% (12/51) of the studies. Most studies (62.7%, 32/51) focused on genes coding for extended-spectrum beta-lactamases (*bla*_ESBl_), which were the most extensively researched (76.5%) CIAs. Additionally, non-beta-lactam resistance genes, associated with aminoglycosides, fluoroquinolones, quinolones, and macrolides (also classified as WHO CIAs for human medicine) were also identified (Supplementary Table 6).

### Origin of AMR *E. coli* in wild birds

Approximately 49% (25/51) of the studies suggested various human activities as possible sources for the AMR *E. coli* found in wild birds (Supplementary Table 4). These suspected sources were linked to landfills (2%, 1/51), contaminated carcasses (3.92%, 2/51), abattoirs (2%, 1/51), polluted water bodies (5.9%, 3/51), sewage effluent (2%, 1/51), nesting areas with high-human densities (2%, 1/51), regions of intensive agriculture where antimicrobials are used in food production (11.8%, 6/51) and unspecified human activities (31.4%, 16/51). These hypotheses were verified in 27.5% (14/51) phylogenetic analyses through sequencing particularly WGS (Haenni et al., 2020; Ong et al., 2020; Fuentes-Castillo et al., 2021; Kurittu et al., 2021; Batista et al., 2022).

### Evidence of wild birds as potential global disseminators of AMR *E. coli*

The absence of satellite-tracking to monitor birds’ movement from their point of origin, stopovers to their final destinations, limited the ability to assess the potential of wild birds in spreading AMR across different regions. Additionally, most studies (96.1%, 49/51) were cross-sectional in design, with only two longitudinal studies (Dreyer et al., 2022; Prandi et al., 2023). Fortunately, phylogenetic relationships in 56.9% (29/51) of the studies were also used to compare genetic relatedness of *E. coli* isolates in wild birds and different locations or other species in distinct locations (Supplementary Table 4).

## Discussion

Antimicrobial resistance *E. coli* has been found in wild birds for nearly five decades, with the first isolation reported in pigeons in 1975 (Sato et al., 1978). This review identified 51 studies examining AMR *E. coli* in wild birds, including both migratory and resident populations. Research remains limited in LMICs except in Nigeria, Egypt, and Bangladesh. Interestingly, despite fewer studies, wild birds in LMICs showed higher levels of AMR *E. coli*, suggesting a link between AMR burden and poor water, sanitation, and hygiene (WASH) infrastructure, poor antimicrobial regulation, and limited healthcare resources. For example, Bangladesh records extensive antimicrobial use in agriculture and minimal waste management (Hasan et al., 2014), while Nigeria lacks proper regulations on antimicrobial use in both human and veterinary medicine (Fashae et al., 2021). In contrast, HICs enforce stricter regulations on AMR use and maintain better WASH systems, contributing to lower AMR levels (Stedt et al., 2014). However, one HIC study recorded a deviation from this trend, where European Starlings from commercial feedlots across five USA states showed high AMR levels (Chandler et al., 2020). Additionally, most studies in both HICs and LMICs focused on non-water birds, probably due to; their proximity to human activities such as landfills, farms, abattoirs, dump sites, agricultural run-offs, urban centers, direct or indirect contact with humans, domestic and companion animals, and their diverse ecological niches and feeding habits.

The presence of phenotypic and genotypic resistant *E. coli* in wild birds across all continents highlights their potential role as reservoirs for AMR and a public health concern for humans, animals, and the environment. A major concern is *E. coli’*s ability to acquire and spread resistances to the WHO CIA List for human medicine. ESBL-producing *E. coli* are particularly problematic as mobile genetic elements (MGEs) facilitate the transfer of these resistances, making *E. coli* both a donor and recipient of AMR in horizontal gene transfer (Guenther et al., 2017). ESBL-producing *E. coli*, once confined to clinical settings, are now increasingly found in wild birds (Guenther et al., 2017). Furthermore, MGEs enable efficient transfer of these resistance across different species and ecosystems (Báez et al., 2015), contributing to the rise of extended-spectrum beta-lactam resistances within the One Health framework (Hasan et al., 2016; Atterby et al., 2017; Fahim et al., 2019; Belmahdi et al., 2022). Migratory birds may further accelerate this spread by carrying AMR genes across regions as white storks migrate between Europe and North Africa (Bouaziz et al., 2018), brown-headed gulls migrate through Tajikistan, Southern China, Pakistan, India, Bangladesh, Myanmar, Sri Lanka, Vietnam and Thailand (Hasan et al., 2014); franklin’s gulls migrate from North America to the Chilean coast (Báez et al., 2015); while different migratory bird species transit the Arabian Peninsula from Africa, Asia and Europe (Elsohaby et al., 2021). Even resident wild birds, though not involved in the long-range AMR spread, can contribute to local transmission to other One Health sectors (Hasan et al., 2016; Oh et al., 2016; Fahim et al., 2019). This calls for preventive and sustainable measures under the One Health framework, guided by the WHO’s GAPs and the Quadripartite in the fight against AMR spread.

Despite increasing reports of AMR *E. coli* in wild birds globally, the origin of these resistant traits remains largely unknown. Only 49% of the studies suggested the potential sources of AMR contamination, and a mere 27.5% verified these hypotheses. The detection of resistant *E. coli* even in wild birds from remote areas (Atterby et al., 2016), further complicates the understanding of transmission pathways. Despite this, numerous studies have established a correlation between high levels of AMR *E. coli* in wild birds and human-impacted environments. These environments include; agricultural fields (Vogt et al., 2018, 2019; Fahim et al., 2019; Blanco and Bautista, 2020; Chandler et al., 2020; Elsohaby et al., 2021; Anueyiagu et al., 2023), dumps (Stedt et al., 2014; Merkeviciene et al., 2018), abattoirs (Fashae et al., 2021), industrial areas (Stedt et al., 2014), contaminated water bodies (Ahmed et al., 2019; Nabil et al., 2020; Yuan et al., 2021) landfills (Bonnedahl et al., 2014), and areas with high human density such as cities (Belmahdi et al., 2022; Tarabai et al., 2023) and beaches (Hasan et al., 2014; Hasan et al., 2016; Mukerji et al., 2019). Fortunately, some sequencing studies have confirmed genetic links between the AMR *E. coli* isolated in wild birds and their hypothesized sources (Supplementary Table 4). This evidence strongly suggests that human activities and their associated environments are major contributors of AMR in wild bird populations. Even in protected areas with minimal human interaction (Machado et al., 2018; Gambino et al., 2021; Suenaga et al., 2019; Yapicier et al., 2022), studies have found that wild birds carry resistant *E. coli* strains. This suggests two main possibilities: either the birds are a natural reservoirs for these resistant strains, or migratory birds are the primary way these strains are being spread to such isolated regions. Further research is vital to fully understand these complex transmission dynamics and develop effective mitigation strategies.

The ubiquity of AMR and their associated genes in the natural ecosystems makes it challenging to trace the exchanges of AMR-associated genes between humans, animals, and the environment, hindering the identification of emerging resistant genes and spread (Vittecoq et al., 2016; Laborda et al., 2022). Our review data indicates wild birds often acquire AMR strains through foraging and direct contact with contaminated environments. Water is considered a key transmission route of AMR to wild birds. This can be through direct consumption of contaminated water or by carrying antimicrobial residues on their feathers or legs in the case of water birds. This phenomenon was observed with aquatic egrets, which transported resistant *E. coli* from the contaminated Jin River to park soil (Wu et al., 2018). These water bodies often receive discharge from domestic wastewater treatment plants (Fashae et al., 2021), hospital and pharmaceutical wastewaters, and agricultural run-offs (Elsohaby et al., 2021). Additionally, wild birds can acquire AMR from agricultural run-offs, which may contain sub-therapeutic concentrations of antimicrobials either in manure or animal feed. This has been demonstrated by the isolation of AMR *E. coli* in migratory birds transiting through Asfar Lake, a large artificial water body formed from the agriculture and livestock drainage water of the earthen drainage network in Saudi Arabia (Elsohaby et al., 2021). Other significant sources of AMR for the birds include untreated landfills (Atterby et al., 2016), dumps, abattoirs, and carcass dumps. These sites often contain human and animal fecal matter and food waste that might be rich in AMR bacteria. It has been suggested that scavengers feeding on livestock carcasses might ingest antimicrobials present in such carcasses (Blanco and Bautista, 2020). Contaminated soil could be a major hot spot of AMR to wild birds and accounts for 30% of AMR genes (Han et al., 2022). As wild birds soak up AMR bacteria from these contaminated sources, they eventually become “ecological sponges,” capable of acquiring and disseminating AMR. The genetic similarities in bird isolates and those in environmental samples (like surface water) further confirm these acquisition pathways (Ahmed et al., 2019; Yuan et al., 2021).

Unfortunately, several research gaps significantly hinder understanding the origin and spread of AMR *E. coli* in wild birds. Our review reveals a stark lack of longitudinal studies, absence of satellite-tracking for studied wild birds, and limited WGS studies. The high cost and time required likely contribute to this scarcity. Despite these challenges, tracing the origins of AMR in wild birds is crucial in informing concerted efforts when developing effective preventive measures within the One Health framework. Future research should therefore prioritize small-scale and longitudinal designs, integration of satellite-tracking, and increased adoption of WGS to enable better tracking of the origins and cross-boundary movement of AMR in wild bird populations and other One Health sectors. By focusing on these areas, researchers can establish a robust foundation for informing more effective and sustainable strategies to limit the spread of AMR in natural ecosystems.

## Conclusion and recommendations

These findings confirm that, indeed, wild birds are potential reservoirs of resistant *E. coli.* The observed bioaccumulation of resistant *E. coli* within these avian populations, coupled with their ability to disseminate AMR across various One Health domains, underscores the urgency to mitigate environmental pollution impacts on AMR. Given that wild birds are not directly subjected to antimicrobial therapeutic interventions, the effective management of AMR in these populations necessitates proactive surveillance facilitated by multi-sectoral collaborations under a comprehensive One-Health framework. This strategic approach aligns intrinsically with the “Zero Pollution Vision for 2050: A Healthy Planet for All.” This vision emphasizes that pollution is a significant catalyst for AMR, and, conversely, pollution abatement is fundamental to ameliorating the AMR crisis. The inherent connection between a healthy environment and the prevalence of AMR is a core tenet of the One Health concept, which acknowledges the indivisible link between human, animal, plant, and environmental health. Therefore, safeguarding avian populations from AMR through environmental remediation will concurrently advance the overarching objectives of One Health and contribute to a healthier planet (European Commission, 2021).

## Data Availability

The original contributions presented in this study are included in this article/Supplementary material, further inquiries can be directed to the corresponding author.
