# Supplementary material for: Wild birds as potential reservoirs of antimicrobial-resistant Escherichia coli: a systematic review
Source: Front Microbiol. 2025 Sep 8;16:1615826. doi: 10.3389/fmicb.2025.1615826 (PMC12450974; doi:10.3389/fmicb.2025.1615826)
Supplement: Supplementary file 1 [file Data_Sheet_1.zip › Supplementary Table 2-Revised.docx]

Supplementary Material

**Table 2.** Percentages of phenotypic resistance patterns of the WHO CIA List in *E. coli* strains isolated from wild birds in different countries

|  |  |  | Highest priority antimicrobials | | | | | | | High Priority antimicrobials | | | | | | | | |
| --- | --- | --- | --- | --- | --- | --- | --- | --- | --- | --- | --- | --- | --- | --- | --- | --- | --- | --- |
| Country | ***E. coli* (n)** | **CAZ** | | **CRO** | **CTX** | **FEP** | **COL** | **CIP** | **NAL** | **MEM** | **CN** | **S** | **AMK** | **AMX** | **AMC** | **AMP** | **ATM** | **Ref** |
| Nigeria | 8 | ND | | 87.5 | ND | 12.5 | ND | 37.5 | 87.5 | ND | 25 | 12.5 | ND | ND | 50 | 75 | ND | (Anueyiagu, et al., 2023) |
| Egypt | 55 | ND | | ND | ND | ND | ND | 75 | ND | ND | ND | 85 | ND | 96 | ND | 82 | ND | (Nabil et al., 2020) |
| Algeria | 3 | 33.3 | | ND | 66.7 | 33.3 | ND | 33.3 | ND | ND | 100 | ND | 0 | 100 | 100 | ND | 33.3 | (Bouaziz et al., 2018) |
| Spain | 79 | 1.3 | | ND | ND | ND | 0 | 6.3 | ND | 0 | 24.1 | 62 | ND | 36.7 | 12.7 | 31.6 | ND | (Blanco and Bautista, 2020) |
| Spain | 38 | 0 | | ND | 0 | ND | ND | 15.8 | 21.1 | ND | 10.5 | ND | 0 | ND | 10.5 | 34.2 | 5.6 | (Martín-Maldonado et al., 2022) |
| France | 88 | NR | | ND | NR | NR | 0 | ND | 13.6 | ND | 39.8 | 25 | 0 | NR | NR | ND | ND | (Haenni et al., 2020) |
| Switzerland | 19 | ND | | ND | ND | ND | ND | 21.1 | 50 | ND | 10.5 | 26.3 | 0 | ND | 5.3 | 50 | ND | (Zurfluh et al., 2019) |
| Germany | 16 | 100 | | ND | 100 | 100 | 0 | 6.25 | ND | 0 | 0 | ND | 0 | 100 | 0 | 100 | ND | (Dreyer et al., 2022) |
| Sweden | 29 | 62.1 | | ND | 100 | NR | 0 | 70 | 55.2 | 0 | 34.5 | 27.6 | 0 | ND | 72.4 | 100 | ND | (Atterby et al., 2017) |
| Lithuania | 179 | ND | | 55 | 15.7 | ND | ND | 47 | 44.6 | ND | 21.5 | 56.9 | 0 | ND | ND | 82 | 100 | (Merkevicieneet al., 2018) |
| Poland | 70 | 8.6 | | ND | 8.6 | ND | 9.6 | 35.7 | 31.4 | 0 | 5.7 | ND | ND | ND | ND | 41.4 | ND | (Skarżyńska et al., 2021) |
| Poland | 32 | ND | | ND | ND | ND | ND | 46.8 | ND | ND | 34.3 | ND | ND | ND | ND | 28.1 | ND | (Nowaczek et al., 2021) |
| Turkey | 51 | ND | | 21.6 | 29.4 | ND | ND | 29.4 | 43.1 | ND | 25.5 | ND | ND | 41.2 | ND | 64.7 | ND | (Yapicier et al., 2022) |
| Saudi Arabia | 90 | ND | | ND | 7.8 | ND | ND | 5.6 | ND | 0 | 12.2 | ND | ND | ND | 8.9 | ND | ND | (Elsohaby et al., 2021) |
| Pakistan | 26 | 100 | | ND | 100 | ND | 3.8 | ND | ND | 0 | 7.7 | ND | ND | ND | ND | 100 | ND | (Mohsin et al., 2017) |
| Bangladesh | 55 | ND | | 18.2 | ND | ND | 10.9 | 50.9 | ND | 7.3 | 10.9 | 74.6 | ND | ND | ND | 100 | ND | (Islam et al., 2021) |
| Singapore | 26 | ND | | 3.8 | ND | ND | 0 | 11.5 | 19.2 | 0 | 7.7 | ND | 0 | ND | 26.9 | 73.1 | ND | (Ong et al., 2020) |
| Australia | 284 | ND | | 62 | ND | ND | ND | 64 | ND | 0 | 18 | 48 | ND | ND | 21 | 86 | ND | (Mukerji et al., 2019) |
| USA | 236 | ND | | 46 | 4 | ND | ND | 10 | 70.5 | ND | 6 | ND | ND | ND | 40 | 85 | ND | (Chandler et al., 2020) |
| Brazil | 49 | ND | | ND | ND | ND | 2 | 0 | 6.1 | ND | 2 | 2 | ND | ND | ND | ND | ND | (Machado et al., 2018) |
| Brazil | 1 | 100 | | 100 | 100 | 100 | ND | ND | 100 | 0 | 100 | ND | 100 | ND | 100 | ND | 0 | (Fuentes‐Castillo et al., 2021) |
| Key: CAZ-ceftazidime; CRO-ceftriaxone, CTX-cefotaxime, FEP-cefepime, COL-colistin, CIP-ciprofloxacin, NAL-nalidixic acid, MEM-meropenem, CN-gentamicin, S-streptomycin, AMK-amikacin, AMX-amoxicillin, AMC-amoxicllin clavulanate, AMP-ampicillin, ATM-aztreonam, ND-not done, NR- not reported | | | | | | | | | | | | | | | | | | |
