# Supplementary material for: Wild birds as potential reservoirs of antimicrobial-resistant Escherichia coli: a systematic review
Source: Front Microbiol. 2025 Sep 8;16:1615826. doi: 10.3389/fmicb.2025.1615826 (PMC12450974; doi:10.3389/fmicb.2025.1615826)
Supplement: Supplementary file 1 [file Data_Sheet_1.zip › Supplementary Table 3 -Revised.docx]

**Table 3**. Percentages of Beta-lactamases and other WHO CIA List genes in *E. coli* strains isolated from wild birds in different countries

| Country | Phenotypic ESBL-*E. coli* producers(n) | AmpC | CP | ESBL | Others^a^ | FQ | AMG | MAC | Ref |
| --- | --- | --- | --- | --- | --- | --- | --- | --- | --- |
| Nigeria | 87 | *^bla^*CMY(2) | 0 | *^bla^*CTX-M-1/15(68), *^bla^*CTX-M-9(17) | *^bla^*SHV(0), *^bla^*TEM(60) | qepA(4) | *strB*, *armA* (1), *ble*(1), *aac6*, aac(6')-Ib *aadA4*(14) | *ermB*(1) | (Fashae et al., 2021 |
| Algeria | 3 | ND | *^bla^*KPC(0), *^bla^*NDM(0), *_bla_*VIM(0), *^bla^*IMP(0) | *^bla^*CTX-M-15 (1) | *^bla^*TEM(3), *^bla^*OXA-48(3) | ND | ND | ND | Bouaziz et al., 2018 |
| Algeria | 8 | ND | ND | *^bla^*CTX-M-14 (8), *^bla^*CTX-M-1 (0), *^bla^*CTX-M-9(0), | *^bla^*SHV(0), *^bla^*TEM(0) | ND | ND | ND | (Belmahdi et al., 2022) |
| Tunisia | 12 | *^bla^*CMY(0) | 0 | *^bla^*CTX-M-15 (8), *^bla^*CTX-M-15+ TEM-1b(4), *^bla^*SHV(0) | *^bla^*OXA (0) | *qnrA1*(8),*qnrB1*(10), *aac-(6′)-Ib-cr*(6) | *aac(3)-II* (10) | ND | Ben Yahia et al., 2018) |
| Spain | 16 | *^bla^*CMY-2 (1) |  | *^bla^*SHV-12 (9), *^bla^*CTX-M-1 (3), *^bla^*CTX-M-14 (2) | *^bla^*TEM-1(5) | *qnrS1*(1) | *aac(6')-Ib* (1), *aac (3)-II(4)* | ND | (Alcalá et al., 2016) |
| Portugal | 7 |  | *^bla^*OXA-1 (3) | *^bla^*CTX-M-15 (3), *^bla^*CTX-M-1(1), *^bla^*SHV-12(2), *^bla^*SHV-55(2) | *^bla^*TEM-1A(1), *^bla^*TEM-1B(5) | *qnrS1*, (2), *qnrB19*(4), *qnrB56*(3), *qnrB67*(3), *qnrB82*(3) | *aac(6’)-Ib-cr*(3), *aac(3)-IIa*(2) | 0 | (Batista et al., 2022) |
| Italy | 22 | ND | ND | 0 | *^bla^*TEM-1(6) | 0 | ND | ND | (Gambino et al., 2021) |
| Greece | 1 | *^bla^*CMY-2 (1) | ND | ND | ND | *qnrA, qnrB, qnrS* | ND | ND | (Athanasakopoulou et al., 2021) |
| Greece | 12 |  | *^bla^*NDM(1) | *^bla^*CTX-M-1(1), *^bla^*CTX-M-1 +*^bla^*TEM(7) | *^bla^*TEM(0), *^bla^*OXA-1 (1), *^bla^*OXA-10(1) | *qnrS*(11), *qnrB*+ *qnrS* (1) | *aadA2* (7), *aadA4* (3), rmtA (5), *aph*A(1) | *mrx(*2), *mph*(9) | (Athanasakopoulou et al., 2022) |
| Switzerland | 5 | 0 | 0 | *^bla^*CTX-M-1(3), *^bla^*CTX-M-15(1), *^bla^*CTX-M-65(1) | 0 | ND | ND | ND | (Zurfluh et al., 2019) |
| Sweden | 29 | 0 | 0 | *^bla^*CTX-M-15 (13), *^bla^*CTX-M-14 (5), *^bla^*CTX-M-55(3), *^bla^*CTX-M-1 (2), *^bla^*CTX-M-32 (2), *^bla^*CTX-M-27 (1), *^bla^*SHV-12 (3) | 0 | ND | ND | ND | (Atterby et al., 2017) |
| Finland | 9 | *^bla^*CMY-2(4), *^bla^*CMY-2+ TEM-32(1) | NR | *^bla^*CTX-M-1(2), *^bla^*CTX-M-15(1) | *bla*CTX-M-15+TEM-1B(1) | *qnrS1*(3) | *aph(6)-Id*(2), *aadA2b*(1), *aph(3")-Ib*(2) | NR | (Kurittu et al., 2021) |
| Lithuania | N/A | *^bla^*CMY-2(1/49) | ND | *^bla^*CTX-M(31/49), *^bla^*PER (0/49) | *^bla^*TEM (38/57), *^bla^*SHV(29/59), *^bla^*OXA-1 (6/56), *^bla^*OXA-3 (10/56), *^bla^*OXA-5 (0/56) |  | *aadB*(0/10), *aadA*(1/10), *rmtB*(0/10), *armA*(0/10), *aphA1*(10/10) *aacA4*(1/10),  *aac(3)II*(5/10), *strA*(20/55), *strB*(21/55) | ND | (Merkeviciene et al., 2018) |
| Poland | 9 | *^bla^*CMY-2(5) | blaOXA-48(1) | *^bla^*CTX-M-15(1) *^bla^*SHV−12(1) | *^bla^*TEM-1B (1),  *^bla^*CTX−M−15 +*^bla^*OXA−1(1) | *qnrS1(4), qnrB19(1)* | *aac(3)-IIa(2), aac(3)-IId(2), ant(3”)-Ia(18), aph(6)-Id(13), aph(3′′)-Ib(13), aac(6′)-Ib-cr(1)* | *mef(*B*)(1),mph(*A*)(1) lnu*G(1) | (Skarżyńska et al., 2021) |
| Poland | 9 | ND | ND | ND | *^bla^*TEM(9) | *qnr*(0) | *aphA1*(6), *aac(3)-IV*(0), *aac(3)-II*(0*),aphA2*(0) | ND | (Nowaczek et al., 2021) |
| Saudi Arabia | 90 | ND | ND | *^bla^*CTX-M-1 (3), blaCTX-M-15(4), *^bla^*CTX-M+TEM (5), *^bla^*CTX-M +SHV(1) | *^bla^*TEM(5), *^bla^*SHV(1) | *qnr*(4) | *aadA1*(0), *aac(3)-v*(8) | ND | (Elsohaby et al., 2021) |
| Pakistan | 26 | NR | NR | *^bla^*CTX-M-15(24), *^bla^*CTX-M-1+TEM-1-C(2) | *^bla^*TEM-1(19) | *qnrS1*(17) | *StrA(18),strB(19),aadA1(2), aadA2(2), aadA5(2), StrA-like(2), StrB-like(2), aac(3)-IVa-like (1), aac(3)-IId-like(1)* | NR | (Mohsin et al., 2017) |
| Bangladesh | 29 | ND | ND | *^bla^*CTX-M-15(28), *^bla^*CTX-M-14(1) | ND | ND | ND | ND | (Hasan et al., 2014) |
| Bangladesh | 21 | *^bla^*CMY-2 (19) | ND | *^bla^*CTX-M(17) | ^bla^SHV(7), *^bla^*TEM(21) | *qnrA*(7), *qnrB*(3) | *aadA1*(17) | *ereA*(0) | (Islam et al., 2022) |
| Singapore | 6 | NR | NR | *^bla^*CTX-M-65(1),  *^bla^*TEM-176(1) | *^bla^*TEM-1B(3) | *qnrS1*(3), *oqxB*(1) | *aac(3)-IV*(1), aadA2, *aph(3′)-Ia*(2), *aph(3”)-Ib*(2*), aph(4)-Ia*(1), *aph(6)-Id*(3), *aadA1*(3), *aadA2*(3) | *mdf(A)(*6), *mph(A)(*1) | (Ong et al., 2020) |
| Mongolia | 14 (but only 4 selected for WGS) | NR | NR | *^bla^*CTX-M-24(1), *^bla^*CTX-M-15(1),  *^bla^*CTX-M-14(2) | *^bla^*TEM-1B(2) | *qnrS1*(1) | *StrA*(2),*strB*(2), *aac(3)-IId*(1), *aadA5*(1), *aac(3)-IIa*(2) | NR | (Guenther et al., 2017) |
| Nicaragua | 10 | NR | *^bla^*NDM(0) | *^bla^*CTX-M-15(6), *^bla^*CTX-M-32(2),  *^bla^*CTX-M-2(1), *^bla^*CTX-M-22(1) | NR | ND | ND | ND | (Hasan et al., 2016) |
| Brazil | 1 | 0 | 0 | *^bla^*CTX-M-8(1) | *^bla^*TEM-1B(1) | 0 | *aph(3)-lb*(1), *aph(6)-Id*(1) | 0 | (Fuentes‐Castillo et al., 2021) |
| Chile | 67 | 0 | 0 | *^bla^*CTX-M-15(41), *^bla^*CTX-M-2(13), ^bla^CTX-M-22(11), *^bla^*CTX-M-3(1), *^bla^*TEM-40(5), *^bla^*TEM-198(1) | *^bla^*TEM-1(28) | ND | ND | ND | (Báez et al., 2015) |

**Key:** AmpC-cephalosporinases, CP-carbapenemases, ESBL-extended-spectrum beta-lactamases, a-Corresponds to beta-lactamases that are not classified as ESBL, AmpC, or CP, FQ-fluoroquinolones, AMG-aminoglycosides, MAC-macrolides, ND-not done, NR, not reported
